# Effects of long-term atorvastatin treatment on cardiac aging

**DOI:** 10.3892/etm.2013.1208

**Published:** 2013-07-05

**Authors:** LEI HAN, MINGGAO LI, XIN LIU

**Affiliations:** Aerospace and Diving Medical Center, Navy General Hospital of Chinese PLA, Beijing 100037, P.R. China

**Keywords:** atorvastatin, cardiac aging, myocardial apoptosis, blood lipids, lipofuscin

## Abstract

A number of studies have reported that atorvastatin (AVT) may have an important role in the delay of cardiac aging. However, the mechanism by which AVT affects cardiac aging has not been established. In this study, a series of experiments were performed to investigate the effects of AVT treatment on the cardiovascular system and the associated mechanism. Wistar rats were administered AVT or saline for 4 months. Age-related changes in the hearts were measured at the end of the experiment. The results showed that compared with young rats, the aged rats had significant changes indicative of myocardial aging, including increased blood lipid 1evelss, increased body weight, cardiac hypertrophy, larger myocardial cells, irregular muscle fibers, fewer deeply stained nuclei, smaller intercellular spaces, a larger number of apoptotic cells and increased levels of lipofuscin in myocardial tissue. However, long-term AVT treatment was able to significantly delay or even reverse these aging-related changes. In addition, these effects showed a certain dose-dependence. In general, long-term AVT treatment reduces blood lipids, inhibits cardiac hypertrophy, suppresses cardiomyocyte apoptosis and lowers the level of oxidative stress to protect the heart from aging.

## Introduction

Human aging is a complex series of specific changes and processes, mainly presenting as organ dysfunction, decreased repair function and the ready occurrence of pathological changes (1–3). Cardiac aging involves a number of senile changes associated with the cardiovascular system and other organs, which affect the structure and function of the cardiovascular system in a similar manner to hypertension, with symptoms including cardiac hypertrophy, reduced ventricular compliance and increased vascular stiffness (4–6).

Myocardial hypertrophy and the reduction of ventricular compliance affect each other within the aging process, and are the pathological basis of diastolic heart failure in the aged (7,8). One of the key factors for improving the quality of life of aged individuals is the delaying of cardiac aging changes, and the subsequent protection of heart function. Therefore, an understanding of the development of cardiac aging and the identification of the underlying molecular mechanisms has become a focus of current medical studies.

Cardiac aging research in recent years has made great progress, which has developed from the overall and organ levels to the cellular and molecular levels (9). For example, left ventricular hypertrophy is one of the most important structural changes in the aging heart (10). A number of variables, including inflammatory factors and peroxisome proliferator-activated receptors (PPARs), have been identified as being important in the aging process of the heart (11,12). Statins are currently the most widely used and effective lipid-lowering drugs in medical practice (13). A number of studies have reported that in addition to lipid-lowering effects, statins also have a variety of other functions, including the reduction of inflammation, anti-oxidation, improvement of endothelial function, inhibition of cell proliferation and other cholesterol-independent effects (14–18). Statins are also able to regulate numerous physiological and pathological processes, including myocardial hypertrophy, myocardial interstitial collagen remodeling and oxidative stress (19–21). Therefore, the cardiovascular protective effects of statins are increasingly attracting the attention of scholars.

Atorvastatin (AVT), as a statin, has been reported to dose-dependently reduce the expression of numerous inflammatory cytokines, attenuate the reduction of PPAR expression levels, inhibit cardiomyocyte hypertrophy and improve the ventricular diastolic function in aged hypertensive patients (22–25). Although there have been several studies on the cardiovascular protective effects of statins, the roles and mechanisms of statin intervention in the occurrence and development processes of cardiac aging have been incompletely investigated. In the current study, naturally aging Wistar rats were chosen as research subjects and the regulatory effects of long-term (AVT) intervention on blood lipids, myocardial cell changes and apoptosis, and oxidative stress indicators in aged rats were studied to explore the protective effect of statin intervention on cardiac aging and the mechanism of action.

## Materials and methods

### Materials

Wistar rats were purchased from Weitong Lihua Experimental Animal Technology Co., Ltd. (Beijing, China). AVT was purchased from Pfizer Pharmaceuticals. Ltd. (Beijing, China). The terminal deoxynucleotidyl transferase-mediated dUTP-biotin nick end labeling (TUNEL)assay kit was purchased from Roche (Indianapolis, IN, USA).

### Animal studies

Ninety 20-month-old Wistar rats were randomly assigned into 3 groups in which the rats were treated with AVT (10 mg/kg/day or 1 mg/kg/day) or saline orally for four months. The body weights were measured every week following drug administration. At the end of the experiment, another thirty 3-month-old rats were set as the young control group. The animals were allowed free access to food and water. Throughout the experiment, the animals were maintained on a 12 h light/12 h dark cycle (lights on at 6:00 a.m.) at 22°C. The experiments were performed in accordance with the Declaration of Helsinki of 1975 and approved by the Ethics Committee of Navy General Hospital of Chinese PLA, Beijing, China. All the animals used in the study received humane care.

### Measurement of blood lipids

Blood samples were collected and the biochemical indicators, including triglyceride (TG), total cholesterol (TC), high-density lipoprotein cholesterol (HDL-C) and low-density lipoprotein cholesterol (LDL-C) levels, were measured using an automatic biochemical analyzer (Hitachi 7060, Tokyo, Japan).

### Histological analysis

The treatment of the rat heart for histological analysis was performed as previously described (26). The sections were then stained using standard hematoxylin and eosin (H&E) protocols to investigate the changes in cardiac morphology induce by the treatment with AVT.

### Myocardial apoptosis assay

Myocardial apoptosis was determined by the TUNEL assay. The detailed steps were carried out using the commercial kit (Roche) following the manufacturer’s instructions.

### Biochemical analyses

The isolated rat heart tissues were washed with saline to remove blood. The tissue was weighed and minced in 9-fold of the tissue weight pre-cooled saline. The minced tissue was homogenized in a glass homogenizer and centrifuged at 1,400 × g for 15 min. The supernatant was collected for biochemical analysis. The content of lipofuscin was estimated by fluorescence analysis using the commercially available kits obtained from Nanjing Jiancheng Bioengineering Institute (Nanjing, China) according to the manufacturer’s instructions.

### Statistical analysis

The results are expressed as the means ± SD. Statistical significance was determined using SPSS 11.0 for Windows (SPSS, Inc., Chicago, IL, USA). One-way ANOVA was performed for multiple comparisons followed by Fisher LSD post hoc comparisons. P<0.05 was considered to indicate a statistically significant result.

## Results

### Effect of AVT treatment on the body weights of rats

The effects of AVT on the weights of all groups are listed in Table I. There were no significant differences in average weight among all groups prior to gavage treatment with AVT (P>0.05). After four months of AVT treatment, the average weights of the high-dose and low-dose AVT groups were significantly reduced compared with those of the aging control group (P<0.01 and P<0.05, respectively), and the comparison of results between the two AVT groups showed that the weight loss of the high-dose group was more evident (P<0.01).

### Changes of blood lipid levels in aging rats and the effects of AVT intervention

The blood lipid levels of all groups are listed in Table II. Compared with the young control group, the TG levels of the aging control group were significantly increased (P<0.01). There was no significant difference in the TG levels between the aging control and low-dose AVT group (P>0.05). The TG level of the high-dose group was lower than that of the aging control group, and the difference in levels was determined to be statistically significant (P<0.05). Comparison of the two statin groups showed that the mean TG level of the high-dose group was lower, but the difference was not statistically significant (P>0.05).

The TC levels of the aging control rats were significantly higher compared with those of the young control rats, (P<0.01). The TC levels of the high-dose and low-dose AVT groups were lower than those of the aging control group (P<0.05 and P<0.01, respectively). Comparison of the two statin groups showed that the TC levels of the high-dose group were lower than those of the low-dose group, and the difference was determined to be statistically significant (P<0.05).

The HDL-C levels of the aging control rats were significantly higher compared with those of the young control (P<0.01). The HDL-C levels of high-dose and low-dose groups presented an elevated trend compared with the aging control group, but the difference was not statistically significant (P>0.05). No significant difference was observed between the two statin groups (P>0.05).

The LDL-C levels of the aging control were significantly higher than those of the young control (P<0.01). The LDL-C levels of the two statin groups were significantly lower than those of the aging group (P<0.01). Comparison of the two statin groups showed that the LDL-C levels of the high-dose group were lower with statistical significance (P<0.05).

### Changes in cardiomyocyte size in aging rats and the effects of AVT intervention

**Figure 1:**
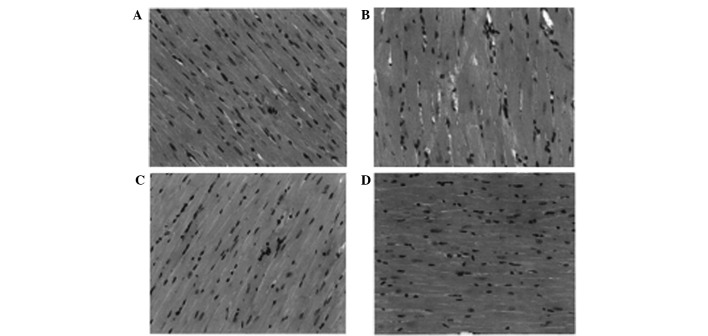
Cardiomyocytes observed by H&E staining (magnification, ×200), cardiomyocyte size (apoptosis) in aging rat was decreased after atorvastatin treatment. (A) Young control group, myocardial cells have the smallest diameter with relatively neat and compact muscle fibers compared with the other 3 groups; (B) aging control group, myocardial cells have the largest diameter and interstitial spaces with the most loose muscle fibers of the 4 groups; (C) atorvastatin low-dose group compared with the high-dose group, the low-dose group has relative large cardiomyocyte size and loose muscle fibers; (D) atorvastatin high-dose group. H&E, hematoxylin and eosin.

The H&E staining results observed under an optical microscope are shown in Fig. 1A. The figure shows that compared with the aged control group, the young rats had myocardial cells with a smaller diameter, a relatively neat and compact arrangement of the muscle fibers, fewer deeply stained nuclei and smaller interstitial spaces between the myocardial cells. The diameter of the myocardial cells was the largest in the aging control group (Fig. 1B), which showed the larger the diameter of myocardial cells, the more serious the extent of apoptosis is, the loose muscle fibers and interstitial spaces also demonstrated the apoptosis of the cells. By contrast, the apoptosis of myocardial cells was decreased following treatment with atorvastatin, and compared with low-dose atorvastatin (Fig. 1C), high-dose atorvastatin (Fig. 1D) was able to inhibit apoptosis more effectively.

The AVT intervention significantly ameliorated cardiomyocyte hypertrophy, reduced the number of deeply stained nuclei and reduced the myocardial interstitial space.

### Impact of aging on myocardial apoptosis and the effects of AVT intervention

**Figure 2:**
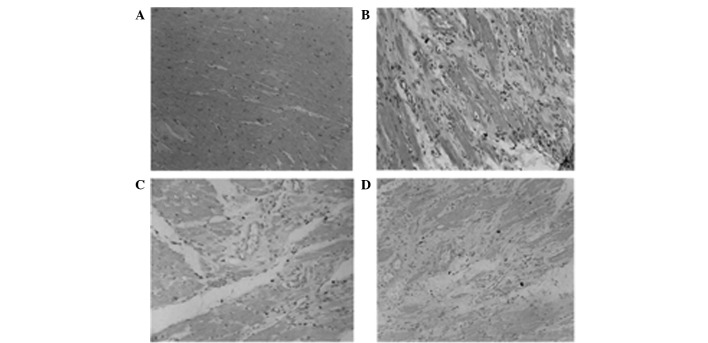
Cardiomyocyte apoptosis observed by TUNEL staining. (A) Young control group, the young rat group have negligible dark nuclei of apoptotic myocardial cells; (B) aging rat group, which has the largest amount of apoptotic (dark) myocardial cells; (C) low-dose atorvastatin group, apoptosis in myocardial cells reduced compared with the aging control; (D) high-dose atorvastatin group, the apoptosis was lower than low-dose group. TUNEL, terminal deoxynucleotidyl transferase-mediated dUTP-biotin nick end labeling.

The results of the TUNEL staining of cardiomyocyte apoptosis are shown in Fig. 2. Under a microscope, the nuclei of apoptotic myocardial cells were dark and the normal cells were gray, the young rat group(Fig. 2A) has negligible apoptotic cells while the aging rat group (Fig. 2B) has the largest amount of apoptotic (dark) myocardial cells. Treatment with atorvastatin reduced the apoptosis in myocardial cells and the apoptosis in the high-dose group (Fig. 2D) was lower compared with the low-dose group (Fig. 2C). The numbers of apoptotic cells are shown in Table III. The apoptotic myocardial cells of the aging control were significantly increased in number compared with those of the young control group (P<0.01). The number of apoptotic cells of the high-dose and low-dose AVT groups were both fewer than those of the aged control group (P<0.01). Moreover, compared with the low-dose group, the reduction of apoptotic cells in the high-dose group was significant (P<0.05).

### Lipofuscin levels of aging rats and the effects of AVT intervention

The lipofuscin levels of the rats are shown in Table IV. The lipofuscin levels of the myocardial tissue in the aging control group were significantly increased compared with those of the young control group (P<0.01). The lipofuscin levels of the high-dose and low-dose AVT groups were both lower than those in the aged control group (P<0.01). Moreover, compared with the low-dose group, the reduction of lipofuscin contents in the high-dose group was significant (P<0.01).

## Discussion

AVT is a statin, which is the most widely prescribed type of cholesterol-lowering medication (27). The effects and the benefits of AVT in regulating blood lipids have been acknowledged by the majority of scholars. Consistent with numerous previous studies, our results showed that AVT played an important role in significantly lowering the lipid levels in aging and young rats, and there were certain correlations between the regulation and the dosage.

In recent years, studies have shown that in addition to lipid-lowering effects, AVT has other independent mechanisms of action, such as the inhibition of vascular smooth muscle cell proliferation (28). According to the results of the current study, the cardiomyocyte size in the aged rat group was markedly different compared with that in the young rats, and after 4 months of long-term intervention with AVT, the average cardiomyocyte diameter of the two AVT groups was significantly lower than that of the aging control group.

The results suggest that the long-term intervention with AVT was able to significantly reduce the myocardial hypertrophy level of aging rats. Numerous previous studies, both *in vivo* and *in vitro*, have confirmed that statins inhibit cardiac hypertrophy by various pathways (29,30). In the present study, the results indicate that the role of AVT in inhibiting cardiac hypertrophy may be associated with the suppression of oxidative stress and inflammatory cytokines.

Apoptosis affects aging in two ways: i) it removes the injured and dysfunctional cells, such as fibroblasts, and replaces them with fibrous tissue to maintain the stability of the body; and ii) it clears the non-renewable cells, such as cardiomyocytes, to cause pathological changes and dysfunction of the body (31). The current study indicates that statins are able to significantly inhibit myocardial apoptosis, thus protecting the heart function. Statins may play important roles in the inhibition of apoptosis by various mechanisms (22).

In the current study, we observed that the myocardial apoptosis levels of the aged rats were significantly increased compared with those of the young rats, which indicated that the increased apoptosis of myocardial cells was an important manifestation of cardiac aging and it combined with cardiac hypertrophy and ventricular remodeling to participate in the pathophysiological process of aging. AVT intervention was able to significantly inhibit the apoptosis of myocardial cells in the aged rats, and the effects in the high-dose AVT group were more marked. The results suggest that AVT may regulate the cardiomyocyte apoptosis signaling pathway, which may be one of the cardiac protective mechanisms of statins.

Lipofuscin has been generally accepted by the majority of scholars to be one of the indicators that reflect oxidative stress, aging state and the effects of anti-aging interventions (32). Previous studies have confirmed that the *in vivo* level of lipofuscin gradually increases with increasing age (33). Therefore, in the present study, we chose lipofuscin as the indicator to reflect the cardiac aging state of the aged rat group and evaluate the effects of the AVT intervention.

According to our experimental results, the lipofuscin levels in the cardiac muscle of the aged rats were significantly increased compared with those of the young rats. Following AVT intervention, the myocardial lipofuscin levels of the aging rats were significantly reduced. The results suggest that AVT may significantly suppress the myocardial oxidative stress level of the aging rats and inhibit the aging of the heart, and suppression of oxidative stress may be one of the cardiac protective mechanisms.

In summary, long-term AVT intervention may reduce blood lipid levels, inhibit cardiac hypertrophy, suppress cardiomyocyte apoptosis and lower the level of oxidative stress to protect the heart from aging.

The cardiovascular systems of the aged rats showed significant aging-related changes compared with those of young rats, mainly presenting as cardiac hypertrophy and markedly elevated blood lipid levels, myocardial apoptosis and lipofuscin levels.

AVT intervention is able to significantly reduce the blood lipid levels of aged rats, relieve myocardial hypertrophy, inhibit cardiomyocyte apoptosis and improve oxidative stress indicators to protect the heart.

## Figures and Tables

**Table I:** Effect of AVT on the body weight of rats (mean ± SD).

Group	Weight (pre-intervention)	Weight (post-intervention)
Young control (n=30)	-	245.83±11.38^a^
Aging control (n=27)	586.67±39.40	632.50±42.77
Atorvastatin low dose (n=24)	581.50±39.46	608.00±41.24^b^
Atorvastatin high dose (n=26)	588.17±39.23	559.17±40.30^a^^c^

a P<0.01,

b P<0.05, compared with the aging control;

c P<0.01, compared with the AVT low-dose group.

AVT, atorvastatin.

**Table II:** Effect of AVT on the blood lipids of rats (mean ± SD).

Group	TG	TC	HDL-C	LDL-C
Young control (n=30)	1.11±0.45^a^	1.89±0.56^a^	1.22±0.37^a^	0.54±0.24^a^
Aging control (n=27)	4.32±2.20	3.55±0.73	1.68±0.42	1.61±0.46
AVT low dose (n=24)	3.54±1.42	3.12±0.35^b^	1.77±0.32	1.16±0.24^a^
AVT high dose (n=26)	3.06±0.85^b^	2.81±0.40^a^^c^	1.82±0.27	0.96±0.25^a^^c^

a P<0.01,

b P<0.05, compared with the aging control group;

c P<0.05, compared with the AVT low-dose group.

AVT, atorvastatin; TG, triglycerides; TC, total cholesterol; HDL-C, high-density lipoprotein cholesterol; LDL-C, high-density lipoprotein cholesterol.

**Table III:** Effect of AVT on cardiomyocyte apoptosis in rats (mean ± SD).

Group	Number of apoptotic cardiomyocyte cells
Young control (n=10)	8.6±5.1^a^
Aging control (n=10)	69.7±25.8
Atorvastatin low dose (n=10)	38.4±19.2^a^
Atorvastatin high dose (n=10)	21.7±13.6^a^^b^

a P<0.01, compared with the aging control group;

b P<0.05, compared with the AVT low-dose group.

AVT, atorvastatin.

**Table IV:** Effect of AVT on the lipofuscin levels of rats (mean ± SD, U/mg·prot).

Group	Lipofuscin level
Young control (n=30)	327.7±39.5^a^
Aging control (n=27)	504.8±40.3
Atorvastatin low dose (n=24)	428.1±33.5^a^
Atorvastatin high dose (n=26)	394.1±29.6^a^^b^

a P<0.01, compared with the aging control group;

b P<0.01, compared with the AVT low-dose group.

AVT, atorvastatin.
